# A Good Antiseptic

**Published:** 1884-01

**Authors:** 


### EDITORIAL.

### A Good Antiseptic.

During the last six months I have had in use a preparation prepared by Lambert & Co., Manufacturing Chemists of St. Louis; to which they have given the name of *Listerine*; into which enter the antiseptic principles of Thyme, Eucalyptus, Baptisia, Gaultheria, Mentha Arvensis and Benzo-Boracic Acid. This constitutes a powerful, safe and pleasant antiseptic, one that is markedly efficient in surgical and dental practice as well. It is unlike some antiseptics that are in general use, in that it is wholly free from offensive odor, and in this respect is rather pleasant than otherwise. It can be used anywhere without injury to fabrics or producing rust. It is used by and has the endorsement of many eminent surgeons in the country.

It is valuable for both internal and external use.

For instance, where there is fermentation and acid eructations from the contents of the stomach; and indeed its disinfectant and antiseptic properties makes it valuable for many affections, especially for those in which fermentation is a factor. For various affections of the throat it is an excellent gargle; it is also valuable as a wash for various conditions of the mouth.

In the treatment of the ordinary diseases of the antrum it is one of the most efficient preparations.

The statement that it is a very effective disinfectant and antiseptic, alike practicable for internal and external uses, and of great value in dental practice, should be sufficient to induce every one to make a trial of it.

Ample directions for its use accompany every package, and the formula as well. I regard it as worthy of a trial by those who desire to have the best things.

### Vick's Floral Guide.

The cultivation of the love for the beautiful, is to be commended in all. A refined taste can nowhere be better displayed, nor with more gratifying results, than in flora culture. The ineffible beauty and endless variety found in plants and flowers, are not equalled elsewhere in nature; and those who avail themselves of the pleasure here to be found, have resources that others little dream of. Any one wishing proof of this need only to carefully look through "Vick's Illustrated Monthly Magazine," the December number of which has just come to hand. This is the Floral Guide for 1884, a beautiful volume, profusely illustrated. The next thing to seeing the flowers is examinnig this guide. It contains 150 pages, with three colored plates of flowers and vegetables, and more than 1,000 illustrations of the choicest flowers, plants and vegetables, and directions for growing. The guide is presented to all the patrons of the house as a Christmas present. All interested in the cultivation of plants and flowers are, or ought, to be thoroughly acquainted with the house of James Vick, Rochester, N. Y. Plants and seeds from this house are entirely reliable, just as represented; of this we have had ample proof in years gone by.

Buy the seeds and the Guide will direct how to grow them. All patrons receive it free, and those who have not been supplied, will receive it by sending 10 cents for postage.

